# Low-Cost Portable Near-Infrared Spectroscopy for Predicting Soil Properties in Paddy Fields of Southeastern China

**DOI:** 10.3390/s26061805

**Published:** 2026-03-12

**Authors:** Minwei Li, Yechen Jin, Hancheng Guo, Dietian Yu, Jianping Qian, Qiangyi Yu, Zhou Shi, Songchao Chen

**Affiliations:** 1 State Key Laboratory of Soil Pollution Control and Safety, Zhejiang University, Hangzhou 310058, China; 3190100129@zju.edu.cn (M.L.); 22414215@zju.edu.cn (Y.J.); ghczju@zju.edu.cn (H.G.); yudietian@zju.edu.cn (D.Y.); shizhou@zju.edu.cn (Z.S.); 2 Zhejiang Key Laboratory of Agricultural Remote Sensing and Information Technology, College of Environmental and Resource Sciences, Zhejiang University, Hangzhou 310058, China; 3 ZJU-Hangzhou Global Scientific and Technological Innovation Center, Zhejiang University, Hangzhou 311215, China; 4 State Key Laboratory of Efficient Utilization of Arid and Semi-Arid Arable Land in Northern China, Institute of Agricultural Resources and Regional Planning, Chinese Academy of Agricultural Sciences, Beijing 100081, China; qianjianping@caas.cn (J.Q.); yuqiangyi@caas.cn (Q.Y.)

**Keywords:** proximal soil sensing, soil spectroscopy, soil spectral library, machine learning, precision agriculture, soil organic matter, paddy soil

## Abstract

Timely and accurate soil property information is critical for sustainable agriculture and precision nutrient management. Conventional laboratory methods are accurate but costly and labor-intensive, restricting their feasibility for high-density soil mapping. Low-cost, portable near-infrared (NIR) spectroscopy presents a promising alternative for rapid, on-site, and non-destructive soil analysis. This study aimed to evaluate the potential of a low-cost, portable NIR sensor (NeoSpectra) for the quantitative prediction of key soil properties in paddy fields from Southeastern China. The target properties were soil organic matter (SOM), total nitrogen (TN), pH, and particle size fractions (clay, silt, and sand). A total of 995 soil samples were collected from representative paddy fields in the region and spectra measurements were conducted in the laboratory on air-dried samples. We developed and compared the performance of multiple machine learning algorithms, including partial least squares regression (PLSR), Cubist, random forest (RF) and memory-based learning (MBL), to build robust calibration models. The predictive models showed substantial performance for SOM and TN, indicating high accuracy (R^2^ > 0.75, LCCC > 0.85, RPD > 2) for quantitative prediction. Predictions for pH, silt, sand, and clay were less accurate (R^2^ of 0.48–0.53, LCCC of 0.67–0.71, RPD of 1.39–1.49), suggesting the sensor’s utility is limited to indicating general trends for these properties. Among the tested algorithms, MBL consistently provided the most accurate and robust predictions across the majority of soil properties. Our findings demonstrate that the low-cost portable NIR sensor, when coupled with appropriate machine learning algorithms, is a powerful and viable tool for the rapid and reliable estimation of critical paddy soil fertility properties (SOM and TN). This technology has significant potential to support field-level soil health monitoring, precision fertilization strategies, and sustainable land management in the agricultural systems of Southeastern China.

## 1. Introduction

Sustainable agricultural management and the advancement of precision agriculture are critically dependent on the accurate and timely assessment of soil properties. Key indicators of soil health and fertility, such as soil organic matter (SOM), total nitrogen (TN), pH, and texture (sand, silt, and clay content), govern nutrient cycling, water retention, and ultimately, crop productivity [1]. Traditionally, the characterization of these properties has relied on conventional wet chemistry laboratory methods. Although accurate, wet chemistry is expensive, slow, and generates hazardous waste, limiting its feasibility for high-density monitoring [2]. This analytical bottleneck hinders the ability of farmers and land managers to make rapid, data-driven decisions for site-specific nutrient management and environmental protection.

To overcome these limitations, diffuse reflectance spectroscopy, particularly in the near-infrared (NIR) region (780–2500 nm), has emerged as a promising alternative for rapid, non-destructive, and cost-effective soil analysis [3]. NIR spectroscopy measures the absorption of light by molecular bonds (e.g., C-H, N-H, O-H) in soil constituents. By applying chemometric and machine learning techniques to the spectral data, it is possible to develop predictive models for various soil properties simultaneously from a single scan [4]. The performance of soil property prediction is heavily reliant on the choice of machine learning algorithm. While partial least squares regression (PLSR) remains a standard linear technique, it often struggles to capture the complex, non-linear relationships present in large, heterogeneous soil spectral libraries [5]. Non-linear algorithms such as random forest (RF) and Cubist have been introduced to address this, yet they still typically apply a global model to the entire dataset. In contrast, memory-based learning (MBL) adopts a local modeling strategy, dynamically selecting spectrally similar neighbors for each prediction sample [5,6]. This approach has shown particular promise in regional-scale studies by effectively handling local spectral non-linearities that global models may over-smooth [6]. Early applications predominantly utilized expensive, laboratory-based spectrometers, which, despite their accuracy, still required soil samples to be transported from the field, thus retaining some of the logistical challenges of conventional methods [7].

Recent technological advancements have led to the miniaturization of spectrometers, resulting in the development of low-cost, portable, and handheld NIR sensors. These devices offer the transformative potential for in situ soil analysis, empowering users to obtain immediate feedback directly in the field [8]. The accessibility and ease of use of these portable sensors are democratizing soil analysis, moving it from the laboratory to the farm. Numerous studies have demonstrated the capability of various portable NIR instruments to predict key soil properties. For instance, research has shown successful predictions of soil organic carbon (SOC), total nitrogen (TN), and soil texture, although the performance can be influenced by factors such as soil moisture and the robustness of the calibration models [9,10].

Among the emerging low-cost devices, the NeoSpectra sensor (Si-Ware Systems) distinguishes itself through the use of Micro-Electro-Mechanical Systems (MEMS) Fourier-Transform (FT-NIR) technology. This design allows for a significant reduction in size and cost while maintaining a wide spectral range (1350–2550 nm) that covers major absorption features for SOM and clay minerals. Unlike discrete-wavelength sensors, the NeoSpectra captures continuous spectral data, making it a potentially transformative tool for soil analysis. Several recent studies have validated its effectiveness across diverse soil types and geographic regions. For example, Sharififar et al. (2019) evaluated the NeoSpectra sensor for predicting soil organic carbon and total carbon, finding its performance to be comparable to more expensive research-grade instruments [11]. Mitu et al. (2024) further investigated the consistency among multiple NeoSpectra units and concluded that while the devices produce comparable spectral data, calibration transfer strategies may be necessary for applications involving multiple sensors to ensure high accuracy [12]. More recently, large-scale soil spectral libraries have been developed using the NeoSpectra sensor, covering a wide diversity of mineral soils in the United States, Africa and Australia, demonstrating its utility for building robust predictive models for properties including SOC, TN, pH, and clay content [13,14]. These studies collectively highlight the growing confidence in the NeoSpectra sensor as a reliable tool for soil science and agronomy.

However, the performance of NIR spectroscopy is highly dependent on the specificity of the calibration dataset, and models developed for one region or soil type may not be directly applicable to another. Paddy soils, in particular, present a unique context due to their management under flooded conditions, which influences their biogeochemical properties. While some studies have explored the use of vis-NIR spectroscopy in paddy soils [15,16,17,18], research focusing specifically on the application of low-cost, portable FT-NIR sensors like the NeoSpectra in this critical agricultural system remains limited.

While low-cost NIR sensors have shown promise, the current literature is heavily skewed toward upland soils in dryland systems. Paddy soils represent a unique challenge due to their distinct mineralogy formed under seasonally flooded conditions, which can obscure spectral features. Furthermore, many prior evaluations rely on relatively small datasets (<200 samples) or standard global calibration models (e.g., PLSR), which often fail to capture regional soil heterogeneity. To date, a comprehensive evaluation of MEMS-based sensors using MBL on a large, regional-scale paddy soil dataset is lacking. Unlike the vast majority of existing low-cost NIR evaluations that focus on stable, aerated upland soils, this study targets the unique complexity of paddy soil systems. These soils undergo periodic redox-driven transformations and anthropogenic compaction (plow pans) that create distinct spectral interference. Consequently, global dryland models are often non-transferable to these critical rice-producing regions, necessitating a regional-scale evaluation that accounts for these specific pedogenic conditions. Therefore, this study aims to address this research gap by evaluating the potential of the low-cost, portable NeoSpectra NIR sensor for predicting key soil properties (SOM, TN, pH, clay, silt, and sand) in paddy soils from Southeastern China. Using a substantial dataset of 995 soil samples along with laboratory spectral measurements on air-dried soil, we will develop and compare the performance of multiple machine learning algorithms to build robust predictive models. The findings of this work will contribute to establishing the utility of this technology for rapid, on-site soil assessment to support precision agriculture in one of the world’s most important rice-producing regions.

## 2. Materials and Methods

### 2.1. Study Area and Soil Sampling

**Figure 1:**
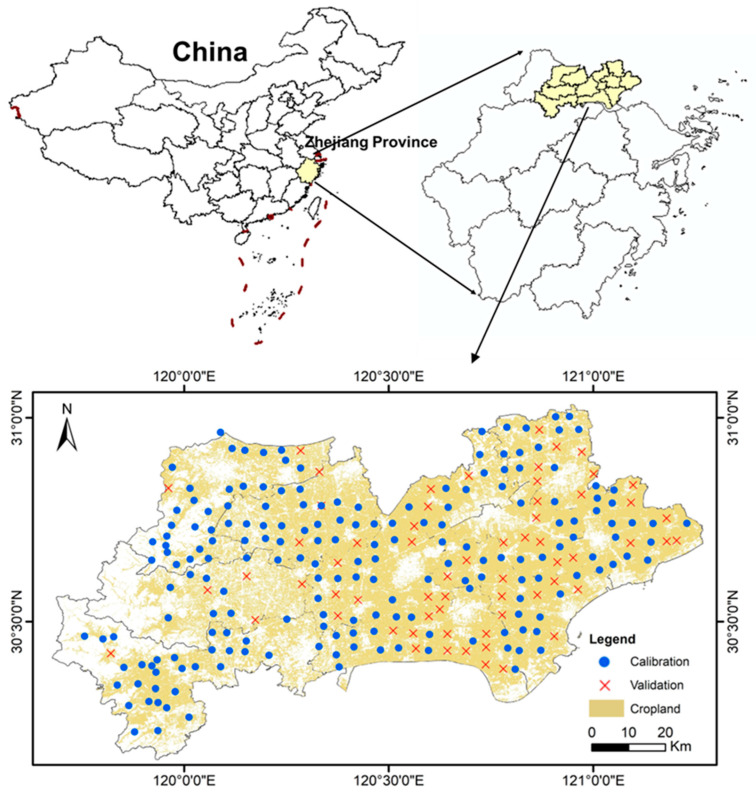
The study area and location of sampling sites for model calibration and validation.

This study was conducted in the Hang-Jia-Hu plain, the largest alluvial plain in Zhejiang Province, China, and a critical component of the Yangtze River Delta (Figure 1). As the province’s most critical grain production base, the region encompasses the major cities of Hangzhou, Huzhou, and Jiaxing, spanning twelve counties and approximately 7600 km^2^. The area is characterized by a subtropical humid monsoon climate, with hot, humid summers and cool winters. The average annual temperature is 16 °C and annual precipitation is approximately 1300 mm. Geomorphologically, the region consists of coastal and lacustrine alluvial plains with low, flat topography, particularly in the east. Rice is the dominant crop, making paddy soils the major soil type in this study area. The cropping system typically follows a rice–wheat or rice–rapeseed rotation, with the rice growing season generally extending from May to October.

Field surveys were conducted from May to June 2025 using a grid-based sampling design stratified by terrain, slope position, soil type, and cropping system. A total of 995 soil samples were collected from 256 sampling sites. At each site, soil cores were taken to a depth of 1 m using a handheld high-frequency vibration soil core sampler (Model VD51, Cote, Melbourne, Australia). The cores were sectioned into four depth intervals (i.e., 0–20, 20–40, 40–60, and 60–100 cm) where possible.

Selected soil physicochemical properties were determined according to standard national protocols. Soil organic matter (SOM) was determined using the potassium dichromate oxidation–external heating method (Walkley–Black). Total nitrogen (TN) was measured using the semi-micro Kjeldahl method. Soil pH was determined potentiometrically using a glass electrode in a 1:2.5 (*w*/*v*) soil-to-water suspension. Soil particle size fractions (clay, silt, and sand) were analyzed using the pipette method after dispersing the soil with sodium hexametaphosphate.

### 2.2. Spectral Measurement and Preprocessing

Spectral measurements were conducted in the laboratory on air-dried soil samples to establish a standardized baseline for the sensor’s performance, eliminating the confounding effects of variable soil moisture and surface roughness typical of field conditions. Samples were air-dried at room temperature until equilibrium moisture content was reached. All samples were ground, and passed through a 2 mm sieve to ensure homogeneity. To ensure the validity of the spectral predictive models, the homogenized soil samples were split into two identical subsamples: one portion was used for the reference chemical analysis (as described in Section 2.1), and the corresponding portion was subjected to spectral scanning. Approximately 100 g of sieved soil was placed into a 10 cm diameter Petri dish, and the surface was carefully leveled using a flat scraper to minimize surface roughness and shadow effects.

Soil reflectance spectra were acquired using a NeoSpectra near-infrared (NIR) spectrometer (Si-Ware Systems, Menlo Park, CA, USA) covering the spectral range of 1350–2550 nm. The NeoSpectra sensor utilizes optical MEMS technology, which significantly reduces cost and enhances portability, making it one of the most widely studied NIR spectrometers in soil science [19]. The instrument operates with a non-uniform spectral sampling interval, averaging a spectral resolution of approximately 8 nm across the wavelength range. For each soil sample, spectral measurements were repeated three times. Each measurement consisted of 10 automatic internal scans. The final representative spectrum for each sample was obtained by averaging the 30 total scans.

To enhance relevant soil information and mitigate noise and scattering effects, several spectral preprocessing methods were evaluated. These included absorbance transformation (AB, AB = log(1/R)), first derivative (FD), and standard normal variate (SNV) correction. In addition to individual methods, combinations were also tested, resulting in a total of six preprocessing strategies (AB, FD, SNV, AB + FD, AB + SNV, and AB + FD + SNV). The optimal spectral preprocessing method was determined by the best model performance for each soil property.

### 2.3. Spectral Modelling

Four machine learning algorithms were employed to predict soil properties from the NIR spectral data.

PLSR is a standard linear technique in soil spectroscopy that addresses multicollinearity by projecting high-dimensional spectral data into orthogonal latent variables (LVs) [20]. This reduces data complexity while maximizing covariance with the response variable. The optimal number of LVs (ncomp) was determined within a range of 1 to 30 by minimizing the root mean square error (RMSE) using 5-fold cross-validation.

RF is an ensemble method that aggregates predictions from multiple decision trees trained on bootstrap samples [21]. The final prediction is derived by averaging the outputs of individual trees. We fixed the number of trees (ntree) at 500 to ensure stability, while the number of features considered at each split (mtry) was optimized between 1 and 15 using 5-fold cross-validation [22].

Cubist is a rule-based piecewise linear model derived from the M5 algorithm [23]. It manages complex non-linear relationships by recursively partitioning the dataset into subsets defined by rules, fitting a separate linear model for each. Two key hyperparameters, committees (10–100) and neighbors (1–9), were optimized via 5-fold cross-validation.

MBL is a local learning approach that avoids training a global model [24]. Instead, it dynamically constructs local regression models for each new sample using spectrally similar cases from the calibration set [25]. In this study, the Mahalanobis distance, calculated on the first two principal components, was used to identify neighbors. A local PLSR model was then fitted for each new sample. The number of neighbors (30–250, step size of 10) and local LVs (3–20) were optimized to balance model stability and accuracy.

### 2.4. Evaluation of Model Performance

**Figure 2:**
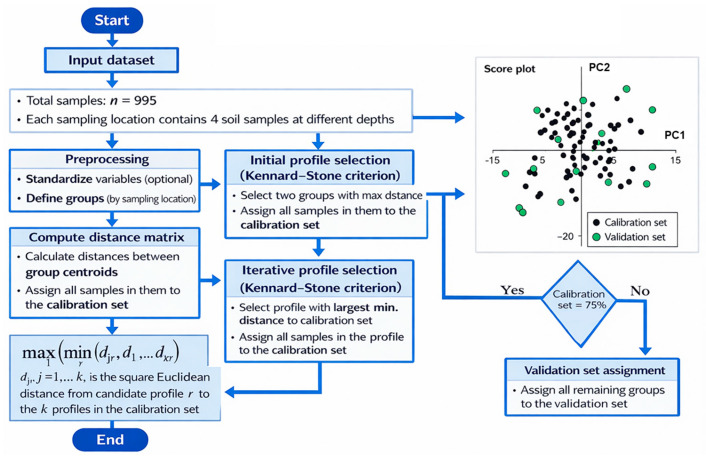
Location-based Kennard–Stone algorithm for splitting the calibration and validation sets.

A total of 995 samples were partitioned into calibration (75%, 746 samples) and validation (25%, 249 samples) sets using a location-based Kennard–Stone algorithm. To prevent over-optimistic model evaluation, the algorithm was constrained to ensure that all samples from a single sampling location (across different depths) were assigned to the same subset. Details of the location-based Kennard–Stone algorithm adopted in this study are illustrated in Figure 2. Model performance of the four algorithms was evaluated on the validation set using the coefficient of determination (R^2^), root mean square error (RMSE), Lin’s concordance correlation coefficient (LCCC), and ratio of performance to deviation (RPD). Higher R^2^ and LCCC values, along with lower RMSE, indicate superior model performance.

## 3. Results

### 3.1. Statistical Summary of Soil Properties and Spectral Characteristics

Descriptive statistics for the measured soil properties are summarized in Table 1. The dataset exhibited a wide range of variation, particularly for soil fertility indicators. SOM and TN showed high variability, with coefficients of variation (CV) of 69.58% and 61.54%, respectively. The soil particle size fractions were dominated by silt (mean of 68.58%), followed by sand (19.85%) and clay (11.57%), reflecting the silt-rich alluvial parent material of the region. Soil pH covered a broad range from acidic (4.4) to alkaline (8.55), with a near-neutral mean of 6.93. Importantly, the statistical characteristics (mean, standard deviation, and range) of the calibration and validation sets were comparable across all properties, confirming that the location-based Kennard–Stone algorithm effectively partitioned the dataset into representative subsets.

**Figure 3:**
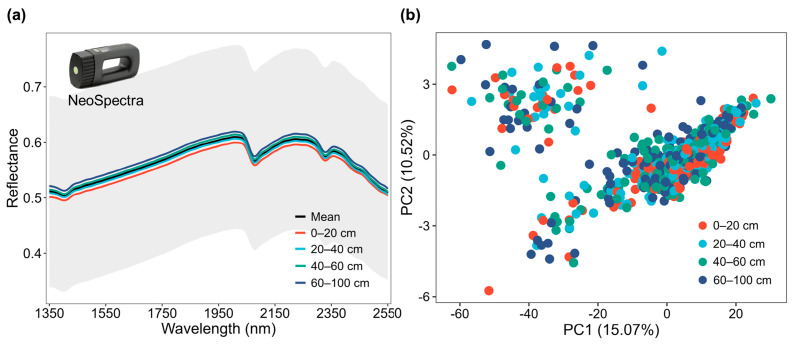
Spectral characteristics of reflectance spectra (**a**) and principal component analysis (PCA) score (**b**) of the collected paddy soil samples using NeoSpectra sensor. In the reflectance spectra plot, the black line represents the global mean, the grey shaded area indicates the standard deviation, and the colored lines correspond to the four sampling depth intervals. In the PCA score plot, the first two principal components (PC1 vs. PC2) illustrate the distribution of soil samples colored by four sampling depth intervals.

The average soil reflectance spectra and their variations across different depth intervals are presented in Figure 3a. The spectra exhibited typical characteristics of soil reflectance in the NIR region, with distinct absorption features near 1400 nm and 1900 nm associated with O-H bonds in soil water and clay minerals, and weaker features around 2200 nm related to Al-OH absorption in clay lattice structures. Overall, soil reflectance increased with wavelength. The standard deviation (shaded area) indicated significant spectral variation among the samples, reflecting the high natural heterogeneity of the soil across the study area. When looking into spectral variation by depth, the topsoil layers (0–20 and 20–40 cm) generally exhibited lower reflectance compared to deeper layers (40–60 and 60–100 cm), likely due to higher SOM in the topsoil, which tends to absorb more light.

The principal component analysis (PCA) performed on the spectral data (Figure 3b) revealed the distribution of soil samples at four depth intervals in the spectral space. The first two principal components (PC1 and PC2) explained 25.59% of the total spectral variance (15.07% and 10.52%, respectively). The score plot illustrates a continuous distribution rather than distinct clustering, reflecting the gradual pedogenic transition down the soil profile. However, a tendency toward separation is observable, where topsoil samples (0–20 cm) generally occupy the lower quadrants (negative PC2 scores) while subsoil samples (60–100 cm) shift toward the upper quadrants, indicating spectral differentiation driven by SOM and texture gradients.

### 3.2. Model Performance Across Different Predictive Models

**Figure 4:**
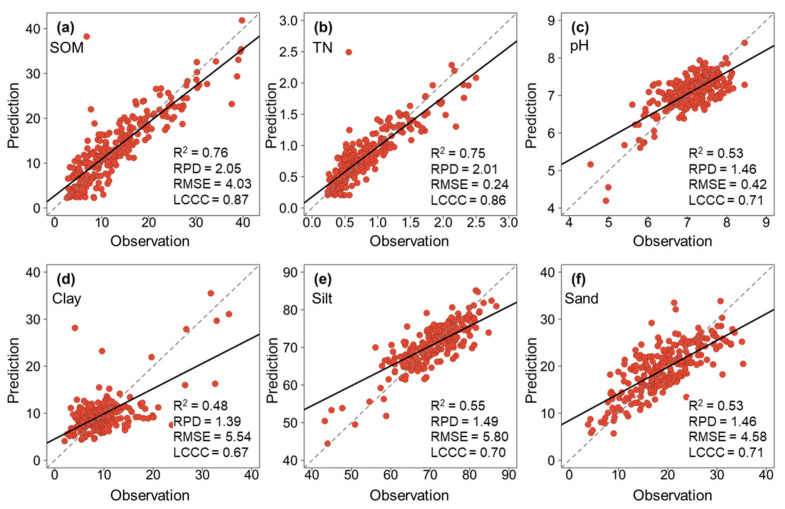
The scatter plots of the best model (MBL) in spectral prediction of SOM (**a**), TN (**b**), pH (**c**), clay (**d**), silt (**e**), and sand (**f**).

The performance of the predictive models for the six soil properties using the validation set is illustrated in Table 2. Among the four machine learning algorithms (PLSR, RF, Cubist, and MBL), the MBL algorithm consistently yielded the most accurate predictions. Consequently, the results presented in Figure 4 focus on the performance of the MBL models.

The models achieved high predictive accuracy for soil fertility properties. SOM prediction was robust, with R^2^ of 0.76, LCCC of 0.87, and RPD of 2.05 (Figure 4a). Similarly, TN predictions showed strong agreement between observed and predicted values (R^2^ of 0.75, LCCC of 0.86, RPD of 2.01) (Figure 4b). These results indicate that the portable NeoSpectra sensor can quantitatively monitor SOM and TN with high reliability. In contrast, the predictions for pH and soil particle size fractions were less accurate. The model for pH achieved R^2^ of 0.53, LCCC of 0.71, and RPD of 1.46 (Figure 4c). For soil particle size fractions, silt content showed the best performance among the fractions (R^2^ of 0.55, LCCC of 0.70, RPD of 1.49), followed by sand (R^2^ of 0.53, LCCC of 0.71, RPD of 1.46) and clay (R^2^ of 0.48, LCCC of 0.67, RPD of 1.39) (Figure 4d–f). While these accuracies are lower than those for SOM and TN, the LCCC values suggest that the models can still distinguish between high and low values, making them useful for rapid screening or identifying general trends.

### 3.3. Model Performance at Different Depth Intervals

**Figure 5:**
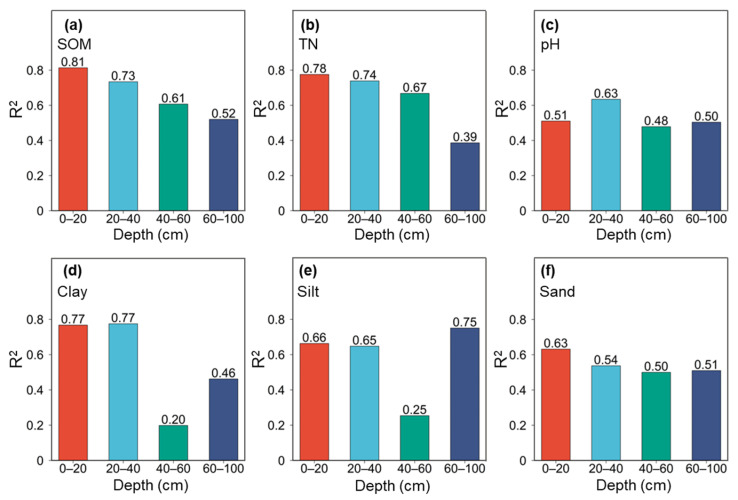
Comparison of model performance in four depth intervals for SOM (**a**), TN (**b**), pH (**c**), clay (**d**), silt (**e**), and sand (**f**).

Model performance across four depth intervals (0–100 cm) revealed clear depth-dependent patterns. SOM prediction was most accurate in the topsoil 0–20 cm layer (R^2^ = 0.81) and 20–40 cm layer (R^2^ = 0.73), but declined noticeably in deeper layers (Figure 5a). However, performance dropped noticeably in the deeper subsurface layers (40–60 cm and 60–100 cm), with R^2^ decreasing to 0.61 and 0.52, respectively. A similar trend was observed for TN, where the 0–60 cm layers exhibited superior predictability (R^2^ of 0.67–0.78) compared to the 60–100 cm layers (R^2^ of 0.39) (Figure 5b). For soil particle size fractions, the depth-wise prediction performance showed more variable patterns. Clay and silt showed an interesting trend that both of them were predicted with relatively high accuracy in the upper layers (R^2^ of 0.65–0.77), followed by a marked decline in the 40–60 cm interval (R^2^ of 0.20–0.25) and a recovery in the deepest layer (R^2^ of 0.46–0.75, Figure 5d,e). In contrast, sand showed a more gradual decrease in predictive performance with depth, with R^2^ decreasing from 0.63 at 0–20 cm to 0.54 at 20–40 cm and remaining around 0.50 in the deeper layers (R^2^ of 0.50–0.51, Figure 5f). These results suggest that the NeoSpectra sensor is particularly effective for assessing key fertility indicators (SOM, TN) and physical properties in the plow layer (0–40 cm), which is the most critical zone for rice root growth and nutrient management.

## 4. Discussion

### 4.1. Potential of NeoSpectra for Characterizing Paddy Soil Properties

This study demonstrates that the low-cost, portable NeoSpectra sensor can successfully predict key soil fertility properties in the paddy fields of Southeastern China. The high prediction accuracy for SOM (R^2^ = 0.76) and TN (R^2^ = 0.75) is comparable to results often achieved with benchtop research-grade spectrometers in paddy soil regions [26,27,28]. This performance is attributed to the direct interaction of NIR radiation with the fundamental molecular vibrations of C–H, N–H, and O–H bonds present in SOM [29]. Given that SOM and TN are the most critical indicators for soil fertility and rice productivity, these results confirm that the MEMS-based NeoSpectra sensor is a viable tool for supporting precision nutrient management in this region.

In contrast, the predictions for pH and soil particle size fractions (clay, silt, sand) were less accurate, serving primarily as indicators of general trends (R^2^ of 0.48–0.55) rather than precise quantitative measurements. This lower performance is consistent with previous studies on portable sensors [12,14]. Unlike SOM, pH does not have a direct spectral response in the NIR region so that its prediction relies on correlations with spectrally active soil components such as clay minerals and SOM [29]. Similarly, while clay minerals have distinct absorption features (e.g., around 1900 and 2200 nm), the complex mineralogy of paddy soils often influenced by redox-influenced iron oxides may complicate the spectral signals. Nevertheless, the achieved accuracy is sufficient for rapid, high-density field screening to identify problematic zones (e.g., acidification or sandy patches) that require further attention.

### 4.2. Superiority of MBL in Regional Modeling

A key finding of this study was the superior performance of the MBL algorithm compared to global modeling techniques like PLSR, RF, and Cubist. Regional soil datasets, such as the one used in this study (covering 7600 km^2^), often present high heterogeneity in parent materials due to pedogenesis as well as human management. Global models like PLSR attempt to fit a single equation to the entire dataset, which often leads to the averaging of spectral features and reduced accuracy for local variations [18,25,30].

MBL overcomes this limitation by dynamically constructing a local model for each unknown sample using only its most spectrally similar neighbors from the calibration library. This approach is particularly effective for complex soil spectral libraries because it implicitly handles non-linear relationships by approximating them with a series of local linear models. Our results suggest that for the regional-scale soil spectral library in Southeastern China, implementing MBL strategies is crucial for maximizing the predictive power of low-cost sensors. The novelty of this approach lies in the integration of MEMS-based sensing with a local modeling strategy (MBL) at a regional scale. While previous studies often utilize small, homogeneous datasets or rigid global models, our results demonstrate that MBL effectively bypasses the ‘averaging’ effect of global equations. This allows low-cost sensors to achieve accuracies (R^2^ = 0.76 for SOM, R^2^ = 0.75 for TN) previously reserved for research-grade benchtop instruments in paddy environments.

### 4.3. Influence of Soil Depth and Management on Prediction Accuracy

The stratification of model performance by depth intervals revealed interesting patterns related to paddy soil pedogenesis and human management. For SOM and TN, predictive accuracy exhibited a clear monotonic decrease with depth. The superior prediction of SOM and TN in the topsoil layers (0–40 cm) aligns with the typical distribution of SOM in cultivated soils, where surface accumulation from crop residues and fertilization creates a wider range of SOC and TN that facilitates robust model calibration. This zone corresponds to the plow layer, which are the most agronomically active and variable layers in rice cultivation systems [31]. Accuracy declined notably in the subsoil (60–100 cm), likely due to the lower concentration and variance of SOM, which reduces the signal-to-noise ratio in the NIR spectra.

In contrast, soil particle size fractions (clay and silt) showed a complex, non-linear trend. While accuracy was high in the surface layer, a marked decline occurred specifically in the 40–60 cm transition zone. This depth often corresponds to the plow pan, a compacted layer characterized by the accumulation of iron–manganese nodules and distinct hydrologic properties due to long-term flooding. These features likely introduce spectral interference that disrupts the correlation between clay minerals and the NIR signal. This identification of a critical depth-dependent limitation provides a necessary boundary condition for portable NIR applications. The disruption of predictability in the 40–60 cm zone is a unique feature of managed paddy profiles, where the accumulation of iron–manganese nodules in the plow pan disrupts the correlation between clay minerals and NIR signals. This finding distinguishes our work from general surface-soil surveys by highlighting how anthropogenic management layers directly impact sensor reliability. The recovery of prediction accuracy in the deep subsoil (60–100 cm) suggests that the parent material at this depth is more homogeneous and free from the distinct anthropogenic disturbances found in the plow pan.

### 4.4. Implications for Precision Agriculture in Southeastern China

The integration of the NeoSpectra sensor with MBL algorithms offers a practical solution to the data bottleneck in precision agriculture. Conventional grid sampling (as performed in this study) is too costly for routine monitoring by smallholder farmers. However, the portability and affordability of the NeoSpectra device enable high-density spatial and temporal measurements, facilitating more effective, data-driven soil monitoring [32,33].

Crucially, to fully leverage the extensive data contained in the China Soil Spectral Library (CSSL), future research must focus on spectral transfer modeling [34,35,36]. The CSSL is primarily constructed using high-precision, research-grade spectrometers (e.g., ASD FieldSpec), which differ significantly in spectral resolution and range from MEMS-based sensors like NeoSpectra. This instrumental discrepancy creates a domain shift that often prevents the direct application of national-scale models to low-cost sensor data. Therefore, developing robust calibration transfer strategies or deep transfer learning is necessary. These methods can mathematically harmonize NeoSpectra readings to align with the ASD standard, effectively bridging the gap between affordable field sensors and high-quality national databases. Successful implementation of this transfer would allow local stakeholders to benefit from the massive, diverse training data of the national soil spectral library without the need for extensive, site-specific recalibration.

While the device currently requires air-dried, sieved samples for maximum accuracy, future work should also focus on calibrating the NeoSpectra sensor for in situ spectral measurements. This will require incorporating external parameter orthogonalization (EPO) or direct standardization (DS) algorithms to remove the interfering effects of soil moisture and surface roughness [15,37]. By combining spectral transfer modeling with in situ calibration, this technology can unlock the full potential of the national soil spectral library, optimizing nitrogen fertilization and soil health monitoring across the Yangtze Delta. Finally, while this study established the feasibility of the NeoSpectra sensor using robust machine learning methods (MBL), future research employing larger-scale libraries (N > 2000) should investigate deep learning approaches (e.g., Convolutional Neural Networks) together with advanced variable selection to potentially capture more complex spectral features [13,38,39].

## 5. Conclusions

This study demonstrates that the low-cost portable NeoSpectra sensor is a viable tool for assessing paddy soil fertility in Southeastern China. MBL proved to be the most robust modeling strategy, consistently outperforming global algorithms. The sensor achieved high predictive accuracy for SOM and TN, making it suitable for quantitative nutrient management, while predictions for pH and soil particle size fractions (clay, silt, and sand) served primarily as indicators of general trends. Furthermore, model performance was superior in the agronomically critical topsoil layers (0–40 cm). Overall, this technology enables cost-effective, high-density soil monitoring to support precision agriculture in intensive rice production systems.

## Figures and Tables

**Table 1:** Descriptive statistics of SOM, TN, pH, clay, silt, and sand. N, number of samples; Max, maximum; Min, minimum; SD, standard deviation; CV, coefficient of variation (%).

Soil Property	Dataset	N	Max	Min	Mean	SD	CV (%)
SOM (g kg^−1^)	Whole	995	68.27	2.27	14.89	10.36	69.58
	Calibration	746	68.27	2.27	15.43	10.84	70.25
	Validation	249	49.89	2.42	13.29	8.57	64.47
TN (g kg^−1^)	Whole	995	3.44	0.2	0.91	0.56	61.54
	Calibration	746	3.44	0.2	0.93	0.58	62.37
	Validation	249	3.17	0.21	0.84	0.49	58.33
pH	Whole	995	8.55	4.4	6.93	0.7	10.1
	Calibration	746	8.55	4.4	6.87	0.72	10.48
	Validation	249	8.45	4.54	7.1	0.61	8.59
Clay (%)	Whole	995	83.74	1.02	11.57	9.4	81.24
	Calibration	746	83.74	1.02	11.97	9.88	82.54
	Validation	249	66.64	2.01	10.36	7.68	74.13
Silt (%)	Whole	995	92.19	9.58	68.58	10.23	14.92
	Calibration	746	92.19	12.12	68.03	10.66	15.67
	Validation	249	86.8	9.58	70.24	8.63	12.29
Sand (%)	Whole	995	53.8	2.83	19.85	7.68	38.69
	Calibration	746	53.8	2.83	20	7.99	39.95
	Validation	249	50.95	3.82	19.4	6.69	34.48

**Table 2:** The coefficient of determination (R^2^), Lin’s concordance correlation coefficient (LCCC), and the ratio of performance to deviation (RPD) for seven soil properties predicted by four models using a NeoSpectra spectrometer. The metrics for the best model are marked in bold font.

Soil Property	PLSR			RF			Cubist			MBL		
	R^2^	LCCC	RPD	R^2^	LCCC	RPD	R^2^	LCCC	RPD	R^2^	LCCC	RPD
SOM	0.59	0.7	1.56	0.72	0.83	1.89	0.74	0.86	1.97	**0.76**	**0.87**	**2.05**
TN	0.57	0.68	1.53	0.73	0.83	1.92	0.74	0.85	1.97	**0.75**	**0.86**	**2.01**
pH	0.46	0.62	1.35	0.49	0.64	1.41	0.5	0.67	1.42	**0.53**	**0.71**	**1.46**
Clay	0.34	0.57	1.23	0.43	0.59	1.33	0.37	0.57	1.26	**0.48**	**0.67**	**1.39**
Silt	0.44	0.55	1.33	0.47	0.62	1.38	0.45	0.62	1.34	**0.55**	**0.7**	**1.49**
Sand	0.26	0.36	1.16	0.44	0.59	1.34	0.5	0.68	1.42	**0.53**	**0.71**	**1.46**

## Data Availability

The data will be available upon reasonable request to the corresponding author.
